# Errata

**Published:** 1886-06

**Authors:** 


### ERRATA.

On page 538, fifth line, for "seyphilis," read "syphilis."

On page 553, tenth line, insert "let" before "the galled jade wince."

In first editorial the types got Dr. Flint's name wrong in the first few copies run off.

Other typographical errors charge up to the hot weather.

